# The predictive value of serum NLR, SII, and OPNI for lymph node metastasis in breast cancer patients with internal mammary lymph nodes after thoracoscopic surgery

**DOI:** 10.1515/biol-2022-0763

**Published:** 2024-04-01

**Authors:** Yang Li, Yang Fei

**Affiliations:** Department of General Surgery, The First Medical Center of Chinese PLA General Hospital, Beijing 100853, China

**Keywords:** systemic immune inflammation index, breast cancer with internal mammary lymph nodes, neutrophil lymphocyte ratio, lymph node metastasis after thoracoscopic surgery, prognostic nutritional index, predictive value

## Abstract

In this research, we delved into the predictive potential of three key markers – the neutrophil-to-lymphocyte ratio (NLR), systemic immune inflammation index (SII), and Onodera’s prognostic nutritional index (OPNI), in assessing lymph node metastases in breast cancer patients who had internal mammary lymph node involvement following thoracoscopic surgery. Our study revealed notable pathological distinctions between the groups with and without metastases, while age, tumor size, and histological grade exhibited no significant differences. The analysis unveiled statistically significant variances in NLR, SII, and OPNI when comparing these two groups. Multivariate analysis pinpointed NLR (OR = 1.503), SII (OR = 1.987), and OPNI (OR = 0.612) as robust predictors of lymph node metastases. Remarkably, combining these markers (AUC: 0.897) substantially enhanced the precision of predicting lymph node metastases compared to individual measurements (NLR: 0.749, SII: 0.717, and OPNI: 0.787). In conclusion, this study underscores the pivotal role of NLR, SII, and OPNI in predicting lymph node metastasis among breast cancer patients with internal mammary lymph node involvement post-thoracoscopic surgery, affirming our utility as reliable independent predictors of this critical clinical outcome.

## 1 Introduction

The incidence of breast cancer has recently surpassed that of lung cancer, rendering it the most prevalent cancer in the world. The internal mammary lymph node, akin to the axillary lymph system, serves as the primary lymphatic drainage location for breast cancer [1]. Lymphatic drainage through the internal mammary lymph nodes is a crucial component of breast cancer therapy. Between 10 and 40% of breast cancer patients are diagnosed with metastases to the internal mammary lymph nodes. A pivotal prognostic factor in breast cancer hinges on the presence of metastasis within the internal mammary nodes (IMN) [2]. The presence or absence of metastasis within the IMN will determine the comprehensive treatment strategy and significantly impact the overall survival rate of patients. Consequently, achieving a definitive qualitative diagnosis of IMN status is imperative.

Lymph node metastasis plays a significant role in predicting postoperative recurrence and metastasis in breast cancer patients. In routine clinical diagnosis, palpation of cervical lymph node metastasis has limited overall efficacy. Although it yields some results, it is associated with relatively high rates of misdiagnosis and missed diagnoses [3]. Non-invasive assessment methods are gaining increasing attention. In recent years, the neutrophil-to-lymphocyte ratio (NLR) and the systemic immune-inflammatory index (SII) in peripheral blood have emerged as easily detectable markers, holding important significance in reflecting systemic inflammatory responses. Numerous studies suggest their utility in assessing the severity of malignant tumors and clinical prognosis in cancer patients [4,5]. Neutrophils are associated with the systemic inflammatory response caused by tumors, while lymphocytes are link to the body’s anti-tumor immune response. Therefore, NLR reflects the dynamic balance between the body’s inflammatory response and its anti-tumor immune suppression level. Indeed, pre-treatment NLR level has been proven to be associated with the prognosis of various tumor patients. The Onodera prognostic nutritional index (OPNI) is a nutritional assessment and surgical risk prediction index established by Japanese scholars. Its advantage lies in its convenience of acquisition and utilization. Preoperative OPNI has demonstrated a close association with the severity of liver, pancreas, and other malignancies [6]. NLR, SII, and OPNI serve as predictive indicators: NLR measures the ratio of neutrophils to lymphocytes, reflecting the balance between inflammation and immune status within the body. SII quantifies systemic inflammation and immune status by combining platelets, neutrophils, and lymphocytes. OPNI is an index that measures the ratio of tumor platelets, neutrophils, and lymphocytes, providing information on tumor driven inflammatory responses and immune depletion. The fundamental principle of these indicators: NLR, SII, and OPNI reflect changes in the body’s inflammation and immune status through the relevant cell types and their ratios in blood samples. High NLR, SII, and OPNI values typically indicate an exacerbation of inflammatory responses and immune system disorders in the body, while lower values indicate better immune status and lower inflammation levels. The objective of this research was to examine the prognostic impact of serum NLR, SII, and OPNI in breast cancer patients who had undergone thoracic dissection, specifically focusing on those with internal mammary lymph nodes. Our goal was to assess the precision and dependability of these indicators in foreseeing the risk of lymph node metastasis and prognosis in breast cancer patients. This study endeavors to introduce fresh perspectives into predicting breast cancer lymph node metastasis, offering valuable insights for clinical practice and therapeutic choices. The results of this investigation carry significant potential for advancing the field and improving the prognosis for breast cancer patients.

## 2 Materials and methods

### 2.1 Material

Breast cancer patients with lymph node metastases in the internal mammary area who underwent thoracoscopic surgery at the Breast Center of the Chinese PLA General Hospital were enrolled in this study. Data were collected from January 1, 2015 to November 30, 2022. Patients were stratified into two groups: those with lymph node metastases after surgery, referred to as the metastasis group (29 patients in total), and those without lymph node metastases, designated as the non-metastasis group (56 patients in total). Inclusion criteria included: (1) breast cancer patients diagnosed by histopathology, (2) the ipsilateral internal mammary lymph node metastasis was confirmed by histopathology, (3) thoracoscopic intramammary lymphadenectomy or modified extended radical mastectomy were performed, and (4) the clinical pathology and follow-up data were complete. Exclusion criteria included: (1) bilateral breast cancer diagnosis, (2) presence of distant metastasis, and (3) history of other malignant tumors.


**Informed consent:** Informed consent has been obtained from all individuals included in this study.
**Ethical approval:** The research related to human use has been complied with all the relevant national regulations, institutional policies and in accordance with the tenets of the Helsinki Declaration, and has been approved by the Medical Ethics Committee of the Chinese PLA General Hospital (No. 2023KY032-KS001).

### 2.2 Methods

(1) General data and laboratory data collection: patient information, including age (>60 years/≤60 years), pathological type, tumor diameter, and histological grade (I/II/III), were collected through self-made questionnaires and electronic medical records. (2) The blood biochemical indexes collection: blood biochemical parameters were collected from patients within 1 week before the operation. The absolute values of serum albumin level, neutrophil (n), lymphocyte (L), and platelet (P) were recorded. NLR and SII were calculated, respectively, where NLR is the ratio of absolute values of N and L, and the SII calculation formula is P × N/L; OPNI = serum albumin level (g/L) + 5 × L(×10^9^/L). (3) Breast tissue specimen collection and analysis: breast tissue specimens were preserved in a 40% formaldehyde solution and embedded in paraffin for clinical pathological analysis. Immunohistochemical staining and analysis were completed according to the standard procedure. The pathological type, tumor size, histological grade, and intraoperative lymph node metastasis were recorded.

### 2.3 Statistical analysis

The statistical analysis was conducted using SPSS 21.0. For normally distributed measurement data, results are presented as *X̄* ± S and a group *t*-test was employed to compare the two groups. The *χ* ^2^ test was used to analyze the statistical significance between the two numerical groups derived from the count data. Variables that showed statistical significance in univariate analysis were subsequently included in the multivariate analysis, utilizing a logistic regression model. To evaluate the predictive value of the related factors for lymph node metastasis, receiver operating characteristic (ROC) curves were generated. A significance level of (*P* < 0.05) was considered as the threshold for determining statistically significant differences.

## 3 Results

### 3.1 Comparative analysis of clinical data between groups

In this study, we present a comprehensive comparison of clinical data between the two groups under investigation: the non-metastatic group (56 cases) and the metastatic group (29 cases). Age, tumor size, and histological grading, on the other hand, did not demonstrate significant differences between the two groups (*P* > 0.05), suggesting that these factors may have limited predictive value in this context. However, a significant difference in pathological types was clearly observed between the metastatic and non-metastatic groups (*P* < 0.05). Specifically, the metastatic group had a higher proportion of cases with invasive lobular carcinoma compared to the non-metastatic group, indicating that the type of cancer pathology plays a crucial role in predicting lymph node metastasis (Table 1).

**Table 1:** Comparative analysis of clinical data between groups

Variable	Number of cases	Non-metastatic group (*n* = 56)	Transfer group (*n* = 29)	*χ* ^2^	*P*
**Age (years)**					
≤60	49	34	15	0.633	0.427
>60	36	22	14		
**Pathological type**					
Invasive ductal carcinoma	74	52	22	4.898	0.027
Invasive lobular carcinoma	11	4	7		
**Tumor size (cm)**					
≤2	47	34	13	1.951	0.163
>2	38	22	16		
**Histological grading**					
I	4	2	2	0.514	0.774
II	67	45	22		
III	14	9	5		

### 3.2 Comparative analysis of systemic inflammatory indices between the groups

Significant statistical differences were evident when comparing the metastatic and non-metastatic groups with regard to NLR, SII, and OPNI (*P* < 0.05), as shown in Table 2. Specifically, the non-metastatic group exhibited a lower mean NLR (1.93 ± 0.72) compared to the transfer group (2.68 ± 0.93), with a statistically significant difference (*t* = 4.113, *P* = 0.000). Similarly, the non-metastatic group displayed a lower mean SII (254.12 ± 65.38) compared to the transfer group (388.54 ± 75.43), and this difference was also statistically significant (*t* = 8.523, *P* = 0.000). Additionally, the non-metastatic group had a higher mean OPNI (52.26 ± 5.09) compared to the transfer group (48.15 ± 6.27), and this difference reached statistical significance (*t* = 3.257, *P* = 0.002). These results underscore the significant distinctions in systemic inflammatory indices (NLR, SII, and OPNI) between breast cancer patients with and without lymph node metastasis, suggesting the potential utility of these indices as predictive factors for lymph node metastasis.

**Table 2:** Comparative analysis of systemic inflammatory indices between groups

Variable	Non-metastatic group (*n* = 56)	Transfer group (*n* = 29)	*t*	*P*
NLR	1.93 ± 0.72	2.68 ± 0.93	4.113	0.000
SII	254.12 ± 65.38	388.54 ± 75.43	8.523	0.000
OPNI	52.26 ± 5.09	48.15 ± 6.27	3.257	0.002

### 3.3 Multivariate linear regression analysis of factors influencing lymph node metastasis

In a multivariate logistic regression analysis, lymph node metastasis (no = 0, yes = 1) served as the dependent variable, while the factors demonstrating statistical significance in the univariate analysis were considered independent variables. As presented in Table 3, the results reveal that NLR (OR = 1.503), SII (OR = 1.987), and OPNI (OR = 0.612) stand out as significant predictors of lymph node metastasis (*P* < 0.05). These results underscore the importance of these systemic inflammatory and nutritional indices in forecasting the likelihood of lymph node metastasis in breast cancer patients, providing valuable tools for clinicians to assess and manage the condition more effectively. The impact of factors such as pathological type was also assessed, revealing additional insights into potential risk factors for lymph node metastasis. Nonetheless, the identified predictors serve as valuable tools in improving risk assessment and decision-making in the clinical management of breast cancer patients.

**Table 3:** Multivariate linear regression analysis of factors impacting lymph node metastasis

Risk factors	*β*	SE	Ward	OR	95% CI	*P*
Pathological type (invasive ductal carcinoma as reference)						
Invasive lobular carcinoma	1.431	1.208	1.403	4.183	0.391–44.643	0.236
NLR	0.407	0.089	20.960	1.503	1.262–1.789	0.000
SII	0.686	0.337	4.151	1.987	1.026–3.8464	0.000
OPNI	−0.491	0.124	15.680	0.612	0.479–0.780	0.000

### 3.4 Predictive potential of NLR, SII, and OPNI for lymph node metastasis

**Figure 1:**
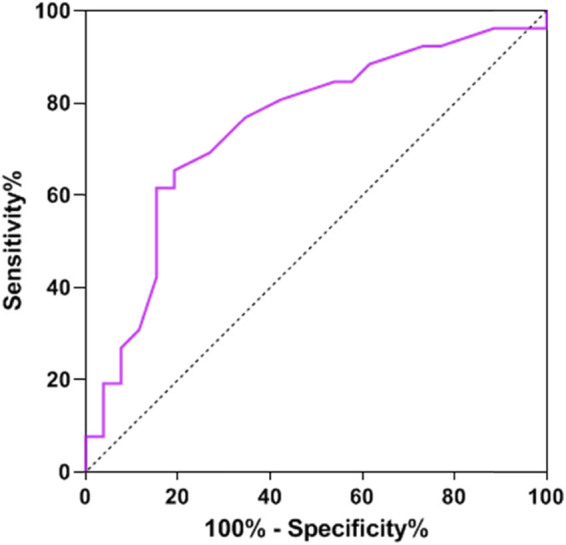
ROC curve of NLR predicting lymph node metastasis.

**Figure 2:**
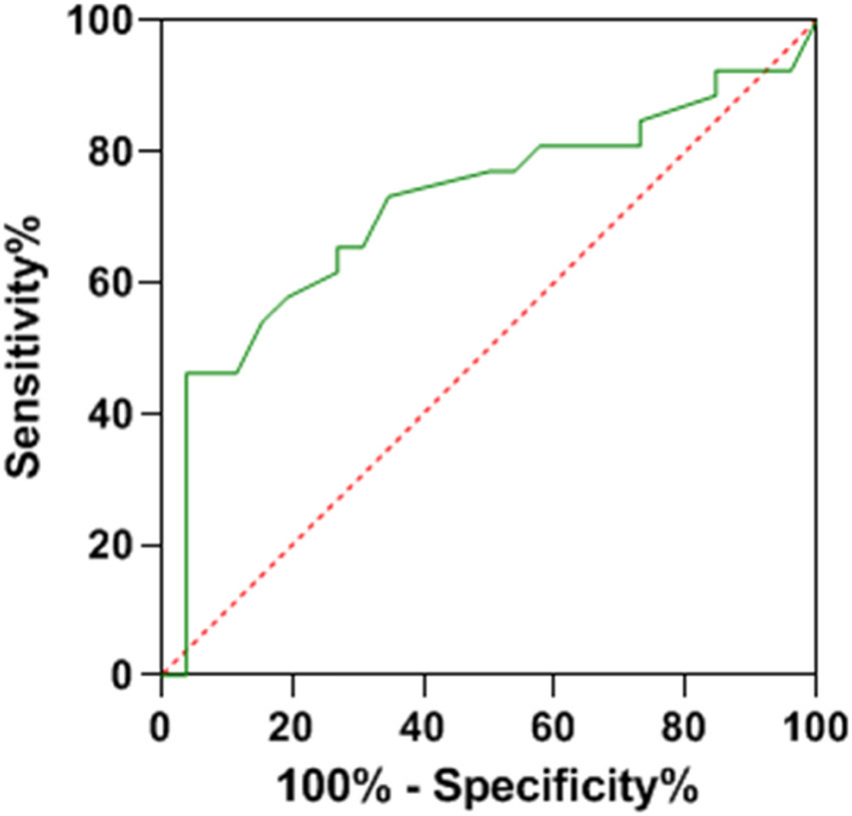
ROC curve of SII predicting lymph node metastasis.

**Figure 3:**
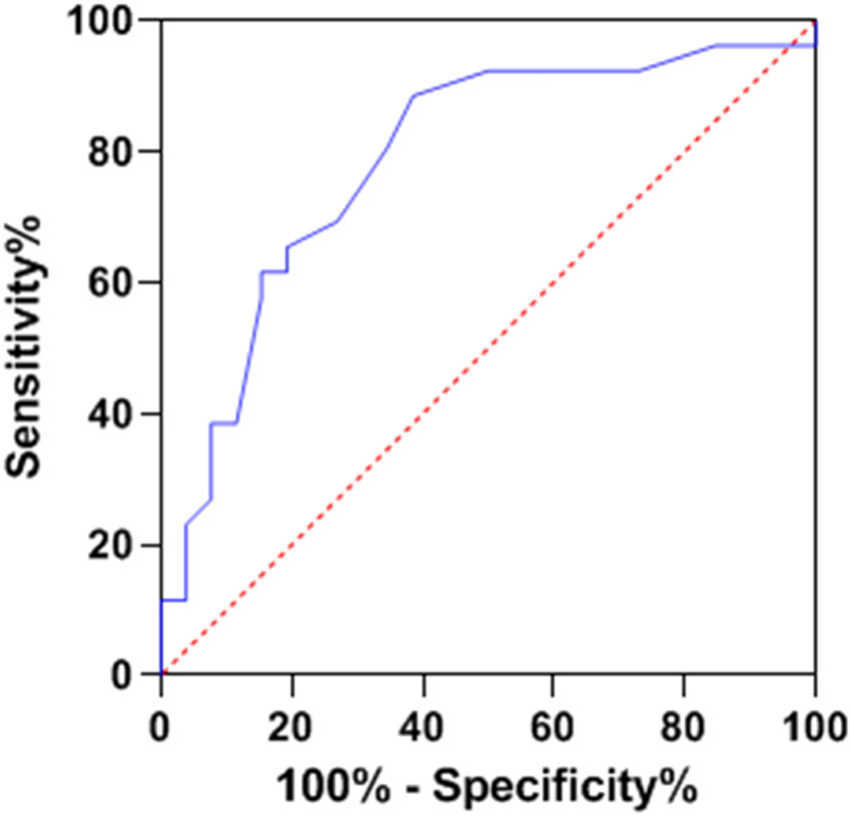
ROC curve of OPNI predicting lymph node metastasis.

**Figure 4:**
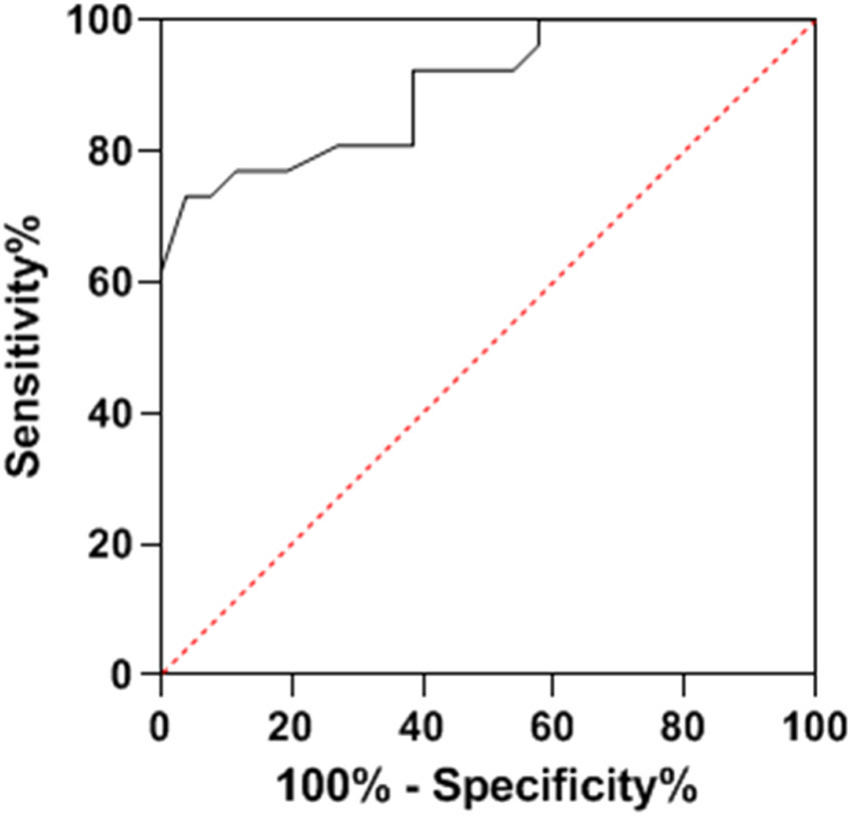
ROC curve of combined detection for predicting lymph node metastasis.

ROC curve analysis provided crucial insights into the predictive capabilities of NLR, SII, and OPNI regarding lymph node metastasis. When evaluated individually, NLR, SII, and OPNI demonstrated area under the curve (AUC) values of 0.749, 0.717, and 0.787, respectively, highlighting their potential to predict lymph node metastases with moderate accuracy. However, the real breakthrough emerged when these three factors were amalgamated, resulting in a significantly improved AUC of 0.897. This impressive AUC for combined detection outperformed the AUC obtained using any single predictor, demonstrating that their synergistic utilization enhances the precision of predicting lymph node metastasis. This novel approach offers healthcare professionals a more reliable and robust tool to assess the risk of lymph node metastasis in breast cancer patients, ultimately guiding more effective treatment strategies and improving patient outcomes. Additional details can be found in Table 4, and Figures 1–4 visually represent the enhanced predictive performance achieved through the joint detection of these indices.

**Table 4:** Predictive potential of NLR, SII, and OPNI for lymph node metastasis

Index	AUC	95% CI	Specificity	Sensitivity
NLR	0.749	0.611–0.886	72.14	69.87
SII	0.717	0.571–0.863	70.87	68.54
OPNI	0.787	0.659–0.915	75.03	73.19
Joint detection	0.897	0.814–0.981	86.19	82.76

## 4 Discussion

Breast lymphatic drainage is primarily categorized into two pathways: axillary and internal mammary. The internal mammary lymph nodes account for draining approximately 25% of breast lymph, while the ipsilateral axillary lymph nodes are responsible for draining the remaining 75% [7]. Previous research has underscored that breast cancer prognosis worsens when it spreads to the internal mammary lymph nodes compared to when it spreads to the axillary lymph nodes [8]. This discrepancy in prognosis exists despite both being regarded as regional lymph nodes of comparable significance. An independent risk variable with a potential impact on a patient’s prognosis is whether their breast cancer has metastasized to the lymph nodes. This study delved into the predictive value of OPNI, SII, and NLR for lymph node metastasis in breast cancer patients with internal mammary lymph node involvement after thoracoscopic surgery. Our findings have unveiled several crucial insights that can guide clinical practice and future research endeavors.

Our study was the notable disparity in pathological types between the metastatic and non-metastatic groups. Specifically, invasive ductal carcinoma exhibited a higher incidence in the metastatic group. This discrepancy highlights the importance of considering pathological types when assessing the risk of lymph node metastasis in breast cancer patients. Previous research has suggested that different pathological types of breast cancer may have varying propensities for lymphatic spread [8]. This emphasizes the need for tailored approaches to lymph node assessment and treatment strategies based on the specific histological characteristics of the tumor.

Breast cancer patients often grapple with malnutrition, primarily stemming from the disease itself and compounded by issues like persistent blood loss. Malnourished individuals facing malignant tumors are at an elevated risk of postoperative complications and typically experience poorer prognoses [9,10]. Malnutrition can adversely affect the immune system and impede cell-mediated immune activity [9,10]. The OPNI, an indicator reflecting nutritional status and immune function, emerged as an influential factor in predicting lymph node metastasis in our study. Serum albumin is one of the commonly used perioperative indicators for the clinical evaluation of patient’s nutritional status and plays an important role in transporting nutrients, maintaining blood permeability, and promoting the formation of repair tissues [11]. The number of lymphocytes is usually an important indicator of the body’s nutritional status and has important significance in reflecting the degree of systemic inflammation and immune status [12]. The OPNI provides a means to gauge patients’ prognoses by assessing their nutritional status and immune function [13]. First, metastasis to lymph nodes can lead to anorexia, digestive tract issues, and malabsorption, all contributing to malnutrition in patients. This, in turn, can compromise immune function and hinder recovery, increasing susceptibility to infections and complications [14]. Second, lymph node metastasis can disrupt the lymphatic system and immune cell function, further impairing immune function and resistance to cancer treatment [15]. Remarkably, our findings have identified OPNI (OR = 0.612) as a key influencing factor in lymph node metastasis. Lymph node metastasis frequently signifies cancer progression and increased malignancy, often leading to a grim prognosis.

In recent years, the role of inflammation in the tumor microenvironment has gained significant attention in cancer research [16]. In our study, we observed that NLR, SII, and OPNI were significantly correlated with lymph node metastasis. These markers reflect the balance between pro-inflammatory and anti-inflammatory factors in the body. Elevated levels of NLR signify an increase in neutrophil count or a relative/absolute decrease in lymphocytes. This rise in neutrophils often signifies tumor growth and metastasis, while a decreased lymphocyte count indicates an abnormal host immune mechanism, typically associated with poor clinical outcomes [17,18]. Similarly, the SII, a comprehensive indicator encompassing peripheral blood lymphocyte, neutrophil, and platelet counts, has emerged as a predictive tool in various cancer types, including pancreatic cancer, hepatocellular carcinoma, small cell lung cancer, and gastric cancer [19]. The combined impact of NLR, SII, and OPNI highlights the intricate interplay between inflammation, immunity, and nutrition in the context of lymph node metastasis.

Our study showed the enhanced predictive accuracy achieved by combining NLR, SII, and OPNI. When assessed individually, these markers demonstrated moderate predictive capabilities, with varying degrees of accuracy. However, their synergy in a joint detection model significantly improved the AUC, surpassing the AUC obtained by any single predictor. This finding underscores the value of integrating multiple biological mechanisms to comprehensively evaluate an individual’s inflammation and immune levels. Moreover, combining these markers enhances specificity and reduces interference from confounding factors, ultimately providing more reliable predictions. The joint detection approach offers healthcare professionals a more robust tool for assessing the risk of lymph node metastasis, aiding in treatment decisions and optimizing patient care.

While our study has provided valuable insights, it is essential to acknowledge its limitations. First, the retrospective design of our study may introduce recall bias and incomplete data, despite our efforts to collect and rectify clinical records. Second, the single-center nature of our study and the limited sample size may introduce selection bias and limit the generalizability of our findings. Future research should involve larger, multicenter studies to validate our results. Additionally, our study did not consider all potential confounding variables, and more complex statistical methods may be necessary in future investigations to address confounding issues comprehensively.

In conclusion, our study sheds light on the predictive value of OPNI, SII, and NLR in breast cancer patients with internal mammary lymph node involvement after thoracoscopic surgery. These markers, reflecting nutritional status, inflammation, and immune function, emerged as significant predictors of lymph node metastasis. Importantly, our combination in a joint detection model substantially improved predictive accuracy. This research contributes to a deeper understanding of lymph node metastasis in breast cancer patients and provides a practical approach to enhance risk assessment and guide treatment decisions. Future endeavors should focus on further validating these findings in larger, more diverse cohorts and exploring the mechanistic links between inflammation, immunity, and nutrition in the context of breast cancer metastasis.
